# Imaging diagnosis of isolated proximal interruption of the right pulmonary artery in a patient presenting with respiratory complications following travel to a high-altitude region

**DOI:** 10.1259/bjrcr.20150071

**Published:** 2015-09-15

**Authors:** Kataveeranahally Shekar Manjunath, Hirennappa B Hudnur, Sengenahally Basavaraja Madhukumar

**Affiliations:** Department of Radiology and Pulmonary Medicine, Columbia Asia Hospital, Bangalore, India

## Abstract

Proximal interruption of the pulmonary artery (PA) is a rare congenital vascular anomaly with an estimated prevalence of 1 in 200,000 young adults. Patients with isolated proximal interruption of the right PA are usually asymptomatic but can present with breathlessness, haemoptysis, recurrent chest infections, pulmonary hypertension or respiratory failure. Such symptoms may be unmasked by pregnancy or at high altitude. We present a case of an isolated interruption of the right PA in a 29-year-old male with a history of cough and breathlessness, requiring hospitalization and ventilator support after travel to a hilly region. Laboratory reports showed normal haemogram and normal renal and liver function tests. Screening test for deep vein thrombosis/pulmonary embolism were negative. Echocardiogram was normal and did not show any evidence of elevated PA pressures. All serial X-rays were reviewed and showed one consistent finding: right lung volume loss with transmediastinal herniation of the left lung to the right side. We discuss the radiological and clinical features along with treatment options for the condition.

## Clinical and laboratory findings

A 29-year-old male presented to the emergency room with sudden onset of dyspnoea and cough that started while travelling in a hilly region. He reported a history of asthma since childhood, requiring the intermittent use of inhalers. There were no other co-morbidities, history of prior tuberculosis or prior severe exacerbations of asthma. He rapidly went into respiratory failure, requiring intubation and high FiO_2_. Chest radiographs revealed left-sided pneumonia and an unexplained right lung volume loss, for which he was treated with antibiotics. He recovered within 3 days and was discharged. He returned for follow-up to understand the cause for his sudden and severe respiratory deterioration at high altitude.

Physical examination was unremarkable apart from the right lung volume loss and a few scattered crepitations on the right side. Laboratory reports showed normal haemogram, and normal renal and liver function tests. D-dimer screening and venous Doppler of both lower limbs were negative for deep vein thrombosis. Echocardiogram did not show evidence of elevated pulmonary artery (PA) pressures.

All prior serial chest radiographs demonstrated a consistent right lung volume loss with transmediastinal herniation of the left lung. High-resolution CT chest, performed the previous year, confirmed right lung volume loss and revealed a subtle reticulation that had been attributed to tubercular sequelae. However, the patient did not know of any prior tubercular illness or antitubercular therapy. He did, however, report that, while *in utero*, his mother had unsuccessfully attempted to medically terminate the pregnancy.

## Imaging findings

**Figure 1.**
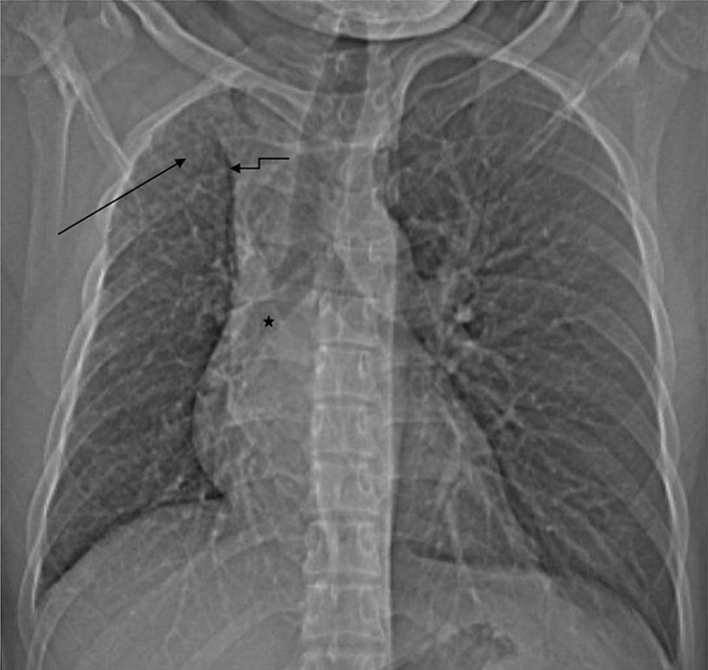
Chest radiograph shows small volume of the right lung, mild elevation of right hemidiaphragm, absence of the mediastinal portion of the right PA (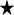), prominent left PA and a shift of the heart, mediastinum and trachea to the right side. The left lung was hyperinflated and herniated over to the right side, as indicated by a displaced anterior junction line (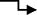). In the small right lung, there are fine reticular opacities peripherally, especially in the upper zone (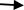). PA, pulmonary artery.

The current chest radiograph demonstrated a small volume right lung, mild elevation of the right hemidiaphragm, ipsilateral mediastinal shift, absence of the right PA, dilated left PA and a hyperinflated left lung that had herniated to the right side, as indicated by a displaced anterior junction line. Within the small right lung, there was a subtle reticulation, most obvious in the periphery of the upper zone. The intrapulmonary vasculature of the left lung was normal (Figure 1).

**Figure 2.**
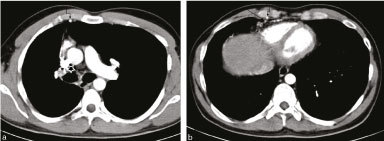
Axial contrast-enhanced CT in the mediastinal window setting revealed prominence of the left PA and its divisions with complete absence of the mediastinal portion of the right PA [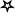 in (a) is the expected location of the right PA]. Epicardial fat contains enlarged, contrast-enhanced intercostal collateral vessels [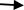 in (b)]. The right internal mammary artery is enlarged [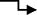 in (a) and (b)]. PA, pulmonary artery.

**Figure 3.**
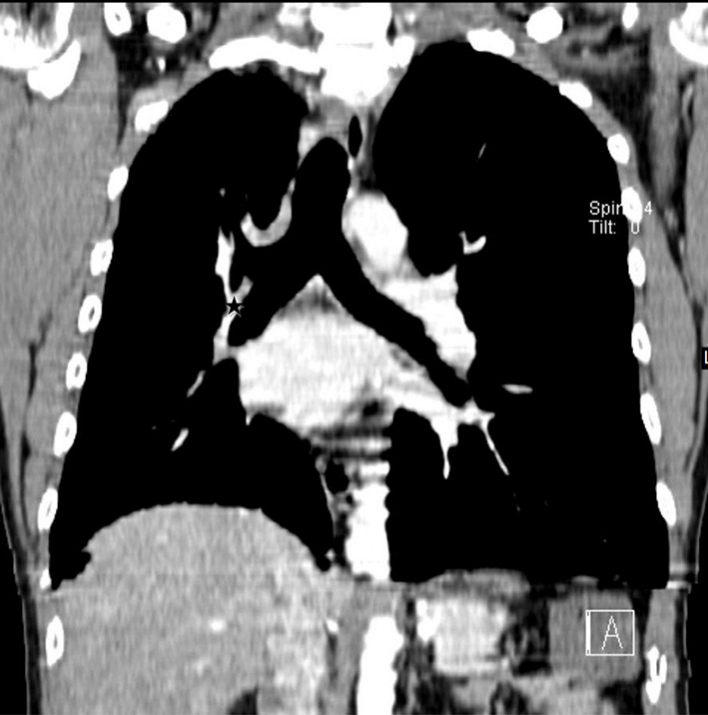
Coronal reformatted images of contrast-enhanced CT chest reveals absence of the mediastinal portion of the right PA (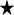 indicates expected location of the right PA). PA, pulmonary artery.

**Figure 4.**
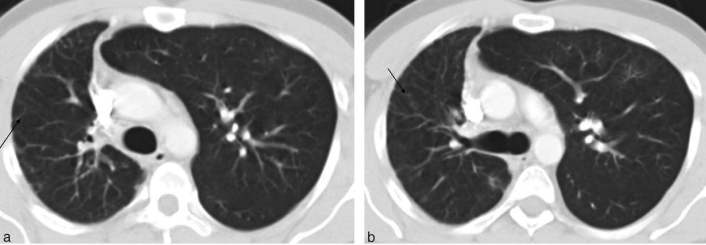
CT scans in lung window setting. The fine reticular opacities within the small right lung represent transpleural collateral vessels [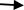 in (a) and (b)]. Herniation of the hyperinflated left lung into the right hemithorax is associated with displacement of the anterior junction line. Although the right lung was small, the bronchial branching pattern was normal and there was no evidence of bronchiectasis, fibrosis or cavity.

Contrast-enhanced CT (CECT) of the chest revealed dilatation of the left PA and its divisions with complete absence of the mediastinal segment of the right PA (Figures 2 and 3). In addition, branches of the dilated intercostal collateral arteries were present in the right epicardial fat (Figure 2b) and a dilated right internal mammary artery was identified in the anterior chest wall (Figure 2a,b). The small right lung demonstrated fine reticular opacification consistent with transpleural collateral arteries (Figure 4a,b). Although the right lung was small, the bronchial airway branching pattern was normal with no evidence of bronchiectasis, fibrosis or cavitation. Herniation of the hyperinflated left lung into the right hemithorax was evident.

## Discussion

Proximal interruption of the right or left PA is a rare vascular developmental anomaly.^1^ The main PAs are derived from the proximal aspect of the sixth aortic arch during the first 16 weeks of intrauterine development.^2^ Although the PA cannot be identified on imaging, careful anatomical dissection will demonstrate a well-developed PA in the lung, with the blind end in the hilum.^3^ Therefore, the term “interruption” is preferred to “absence” of the PA. In the majority of cases, the interrupted PA is contralateral to the side of the aortic arch.^4^

The involved lung is only mildly hypoplastic because the pulmonary arterial tree distal to the interruption remains connected to the patent distal segment of the sixth arch or ductus arteriosus.^5^ Also, there is a collateral arterial supply to the lung that is derived from the systemic circulation, primarily the bronchial arteries, but also the intercostal and internal mammary arteries.^5^ Most of these vessels hypertrophy following birth and the closure of the ductus arteriosus. Most patients with proximal interruption of the right PA are asymptomatic and diagnosed incidentally in adulthood. In contrast, interruption of the left PA is usually associated with other cardiovascular congenital anomalies, such as tetralogy of Fallot, and is therefore diagnosed early.^6^

Chest radiographs demonstrate a smaller volume hemithorax, compensatory hyperinflation of the contralateral lung, elevation of the ipsilateral hemidiaphragm, absent ipsilateral PA, enlarged contralateral PA and ipsilateral shift of the mediastinum.^1^ Prominent and hypertrophied intercostal arteries may result in notching of the ribs.^6^ Intrapulmonary collaterals create a reticular pattern in the lung and subpleural collaterals may cause pleural thickening.

Differential diagnoses for proximal interruption of the right PA are simple pulmonary hypoplasia, hypogenetic lung syndrome (scimitar syndrome), Swyer–James syndrome and tubercular sequelae. Simple pulmonary hypoplasia is often radiographically indistinguishable from proximal interruption of a PA, particularly when the PA is markedly hypoplastic.^7^ Hypogenetic lung syndrome (scimitar syndrome) is a rare form of partial anomalous pulmonary venous return in which an anomalous pulmonary vein is the draining vein for part or the entire entire right lung, emptying into the inferior vena cava, either above or below the diaphragm. The anomalous vein may occasionally drain into the hepatic veins, portal or azygous vein, coronary sinus or right atrium. The anomalous vein is usually associated with various degrees of hypoplasia of the right lung and with a hypoplastic or aplastic PA. Chest radiographs may show a characteristic scimitar-shaped shadow, representing the anomalous draining vein. CT angiography is the non-invasive method of choice for diagnosing hypogenetic lung syndrome.^8^ In the Swyer–James syndrome, a hyperlucent small or normal-sized hemithorax is seen on the affected side with evidence of air trapping on expiratory radiographs. In proximal interruption of a PA, the small lung may occasionally appear hyperlucent and mimic the Swyer–James syndrome,^4^ but more often, the affected lung is minimally denser than the contralateral lung,^9^ because it contains fewer and smaller alveoli related to exposure to desaturated blood, an important stimulus for alveolar development in the first 8 years of life.^9^ In proximal interruption of the PA, regardless of how radiopaque the affected lung is, the bronchial tree is also normal with no evidence of air trapping or bronchiectasis.^10^ The reticular pattern may be confused with post-inflammatory changes and fibrosis but unlike in proximal interruption of the PA, post-tubercular sequelae do not include a marked discrepancy in the size of the hila.

Haemoptysis occurs in approximately 10% of cases of interruption of the PA owing to rupture of thin-walled, hypertrophied collateral vessels.^11^ Such episodes of haemoptysis are usually minor and self-limited, but massive haemoptysis may rarely occur, requiring emergency treatment by means of pneumonectomy or embolization of systemic collateral vessels.^11^

The definitive diagnosis of this condition can be made by angiography or by non-invasive CECT, the preferred technique.^12^ Invasive angiography is reserved for cases requiring embolization for control of haemoptysis. In our case, the diagnosis was made on a CECT. Absence of the mediastinal portion of the PA and presence of a small hilar segment of the affected PA are both well demonstrated on CECT. CECT can also demonstrate the contrast-enhanced collateral vessels^12^ and allow concurrent evaluation of the bronchial tree and the lung parenchyma, in addition to the major vascular structures. The fine reticulation owing to collateral vessels within the lung on the side of the interrupted PA and the normal bronchial tree pattern are demonstrated on CT lung windows.

There is no consensus regarding the treatment of isolated proximal interruption of the PA. Most agree that treatment should be reserved for the small number of patients with haemoptysis, recurrent lower respiratory tract infections or pulmonary hypertension. Treatment options include partial or total pneumonectomy, closure of selected collateral arteries not solely responsible for pulmonary blood flow, or a primary versus staged PA anastomosis. In the asymptomatic patient with isolated proximal interruption of the PA, conservative management with close follow-up seems most appropriate.

## Learning points

1. Proximal interruption of the right or left PA is an uncommon anomaly.
2. In most cases, the interrupted PA lies on the opposite side of the aortic arch.
3. The collateral supply of the involved lung is derived from the systemic circulation, mainly the bronchial arteries, intercostal and internal mammary arteries.
4. Most patients with proximal interruption of the right PA are asymptomatic. In contrast, interruption of the left PA is usually associated with other cardiovascular congenital anomalies. most commonly tetralogy of Fallot.
5. Chest radiographs demonstrate decreased size of the affected hemithorax with compensatory hyperinflation of the contralateral lung.
6. Simple pulmonary hypoplasia, hypogenetic lung syndrome (scimitar syndrome), Swyer–James syndrome and tuberculosis are the radiographic differential diagnoses.
7. The definitive diagnosis of this condition can be made by angiography and CT. CT, being non-invasive, is preferred with invasive angiography reserved for cases of haemoptysis requiring embolization.
8. CT enables concurrent evaluation of the bronchial tree and the lung parenchyma. Absence of the mediastinal portion of the PA, presence of a small hilar segment of the affected PA and prominence of the contrast-enhanced collateral vessels are demonstrated by CT.
9. There is no consensus regarding the treatment of isolated proximal interruption of PA. Most agree that treatment should be reserved for the small number of patients with haemoptysis, recurrent lower respiratory tract infections or pulmonary hypertension.
